# Effectiveness of CAR-T treatment toward the potential risk of second malignancies

**DOI:** 10.3389/fimmu.2024.1384002

**Published:** 2024-05-02

**Authors:** Massimo Martino, Gaetana Porto, Giorgia Policastro, Caterina Alati, Barbara Loteta, Maria Caterina Micó, Clizia Argiró, Maria Altomonte, Tiziana Moscato, Demetrio Labate, Vincenzo Dattola, Carmelo Massimiliano Rao, Francesca Cogliandro, Filippo Antonio Canale, Virginia Naso, Gianfranco Filippelli, Antonino Iaria, Martina Pitea

**Affiliations:** ^1^ Stem Cell Transplantation and Cellular Therapies Unit (CTMO), Department of Hemato-Oncology and Radiotherapy, Grande Ospedale Metropolitano “Bianchi-Melacrino-Morelli,”, Reggio Calabria, Italy; ^2^ CAR-T Multidisciplinary Team, Grande Ospedale Metropolitano “Bianchi-Melacrino-Morelli,”, Reggio Calabria, Italy; ^3^ Hematology Unit, Department of Hemato-Oncology and Radiotherapy, Grande Ospedale Metropolitano “Bianchi-Melacrino-Morelli”, Reggio Calabria, Italy; ^4^ Pharmacy Unit, Grande Ospedale Metropolitano “Bianchi-Melacrino-Morelli”, Reggio Calabria, Italy; ^5^ ICU Unit, Grande Ospedale Metropolitano “Bianchi-Melacrino-Morelli”, Reggio Calabria, Italy; ^6^ Neurology Unit, Grande Ospedale Metropolitano “Bianchi-Melacrino-Morelli”, Reggio Calabria, Italy; ^7^ Cardiology Unit, Grande Ospedale Metropolitano “Bianchi-Melacrino-Morelli”, Reggio Calabria, Italy; ^8^ Oncology Department, Hospital of Paola, Cosenza, Italy; ^9^ Oncology Unit, Melito Porto Salvo, Reggio Calabria, Italy

**Keywords:** car-t, follow-up, second cancer, malignancies, therapy

## 1 Introduction

Chimeric antigen receptor-modified T-cell (CAR-T) is a clinical and technological revolution (1, 2). The more advanced developments that led to the commercialization of the first products, have identified as a target the molecule CD19, expressed in all leukemia acute lymphoblastic leukemia (ALL) (3) and in Non-Hodgkin Lymphoma (NHL) (4), such as diffuse (D) large B-cell lymphoma (LBCL) (5), follicular lymphoma (FL) (6), mantle-cel lymphoma (MCL) (7) and the molecule B-cell maturation antigen (BCMA), expresses in multiple myeloma (MM) (8, 9). This approach has opened a new page in medicine because we have moved from the drug, understood as an active ingredient packaged and ready to be taken, to a highly personalized therapy. Results from CAR-T trials are showing unprecedented outcomes in patients with no other treatment options beyond palliative care (10).

As cellular products, CAR T cells are associated with unique toxicities, and cytokine release syndrome (CRS), immune effector cell-associated neurotoxicity syndrome (ICANS), and cytopenias have been a challenge that has involved a major commitment to the entire scientific community (11). With a growing experience, management of these toxicities is an evolving field, and the current management strategies include continuous monitoring of the patient in the first thirty days post-infusion, rapid detection, and accurate intervention with supportive care, anti-cytokine or corticosteroid therapy. Other toxicities, rarer but described, are an infusion reaction, tumor lysis syndrome, anaphylaxis and immunogenicity, B-cell aplasia and hypogammaglobulinemia, infections and hemophagocytic lymphohistiocytosis/macrophage activation syndrome (12–14).

Data on late toxicities of CAR T cells, including effects on the immune system, such as a new occurrence or exacerbation of neurologic or autoimmune disorders, and secondary malignancies require longer observation periods. The possible development of second cancers is a price to pay for many “lifesaving” treatments, in this setting of patients. The increased risk of second malignancies is related to a combination of factors, including molecular background, host immunological status, genetic predisposition, and chemotherapy administered (15). Second cancers constitute 15 to 20% of all cancer diagnoses in the cancer registries, and the risk of a second cancer is significantly higher than in the general population, between 3- and 10-fold (16).

Although a follow-up of 15 years has been requested as part of the marketing authorization of commercially available CAR T cells, the European Medicines Agency announced on January 12, 2024, that its Pharmacovigilance Risk Assessment Committee has beginning a review of data on secondary malignancies related to T-cells for approved CAR-T therapies (17).

In this paper we evaluated publications pertaining to follow-up updates of clinical trials, specifically presented at the 2023 ASH congress, with a focus on incidence of second cancers (**Table 1**).

**Table 1:** Description of second malignancies in studies with updated follow-up.

Study	Characteristics	Disease	No. Patients	Secondary malignancy	Median Follow-Up
ZUMA-7 Ref. 27	First randomized, global, multicenter, Phase 3 study of axi−cel versus standard of care as second−line treatment in patients with, axi−cel showed significantly improved event−free survival (EFS) compared with second−line SOC (hazard ratio [HR], 0.398, P<.0001; median 8.3 versus 2.0 months; 24−month EFS rate: 41% versus 16%	Early R/R LBCL	Axi−Cel, ≥65 Years N=51 SOC, ≥65 Years N=58	Axi−Cel, ≥65 Years 1 (2%) Acute myeloid leukemia SOC, ≥65 Years 0 (0%)	46.6 months
ZUMA-12 Ref. 29	Phase 2, multicenter, open-label, single-arm study of axi-cel as part of first-line treatment. In the primary efficacy analysis (n=37; median follow-up of 15.9 months), axi-cel demonstrated a high rate of durable responses with an investigator-assessed CR rate of 78% (and an ORR of 89%	high-risk LBCL	37	1 esophageal adenocarcinoma,	40.9 months
Real-world Ref- 31	Commercial use of liso-cel based on a postmarketing study using data collected at the Center for International Blood and Marrow Transplant Research (CIBMTR)	R/R LBCL	396	Squamous cell skin malignancy 5 (1%) Myelodysplasia 3 (1%) Basal cell skin malignancy 2 (< 1%) Gastrointestinal malignancy 2 (< 1%) Melanoma 1 (< 1%) Myeloproliferative neoplasm 1 (< 1%)	
PILOT Ref. 35	Open-label phase 2 study evaluated the efficacy and safety of liso-cel in patients not intended for HSCT after 1 prior line of therapy. In the primary analysis, the primary endpoint was met with an ORR of 80%	R/R LBCL	61	2 (4%) Squamous cell carcinoma of skin and malignant external ear neoplasm (n = 1) Myelodysplastic syndrome (n = 1)	18.2 months
Elara Ref. 38	Phase II, single-arm, global, multicenter, open-label trial investigating the efficacy and safety outcomes of tisagenlecleucel in adults after ≥2 treatment lines or who relapsed after autologous stem cell transplant (autoSCT)	r/r FL	97	2 (squamous cell carcinoma and bladder transitional	29.0 months
ZUMA-5 Ref. 40	Axi-Cel; Single-arm, registrational, phase 2 trial ≥18 years; ≥2 prior systemic therapies that must have included an anti-CD20 monoclonal antibody combined with an alkylating agent.	R/R iNHL, including FL (grade 1-3a) and MZL (nodal or extranodal;	159 (127 FL, 31 MZL, 1DLBCL)	5 (unknown origin, unrelated to axi-cel)	36 months
CARTITUDE-1 Ref. 50	Single-arm, open-label, multicenter, phase Ib/II study conducted in patients to characterize the safety of cilta-cel and confirm the recommended phase II dose (phase Ib) and evaluate clinical efficacy	RRMM	97	20 secondary primary malignancies were reported in 16 patients; all were unrelated to cilta-cel. 9 hematologic SPM, including 1 low-grade B-cell lymphoma, 6 myelodysplastic syndrome, and 3 cases of fatal acute myeloid leukemia (AML; 1 patient had both myelodysplastic syndrome and fatal AML) 4 patients had squamous cell carcinoma; 1 of these also had basal cell carcinoma. 1 patient had basal cell carcinoma that was present before cilta-cel infusion. 1 patient each had malignant melanoma, adenocarcinoma, or myxofibrosarcoma, and 1 patient had prostate cancer in addition to his squamous cell carcinoma and AML reported above.	27.7 months
LEGEND-2 Ref. 51	phase 1, single-arm, open-label study	RRMM	74	2 lung cancers at 8 and 32 months 1 esophageal cancer at 15 months 1 Cervical cancer at 8 months, after the CAR-T cell infusion	47.8 months

### 1.1 ALL

Anti-CD19 CAR-T cell therapy is approved for patients up to 25 years of age and for adult affect by ALL based on the ELIANA (tisagenlecleucel-tisa-cel) (18) and ZUMA-3 trial (brexucabtagene autoleucel – brex-cel) (19). Most trials of CAR-T cells in relapsed/refractory (RR) ALL demonstrate impressive response rates, with >70% of patients achieving complete remission (CR) (20). Given the poor prognosis, there appears to be no evidence of secondary malignancy in this setting, where we emphasize there are no therapeutic alternatives.

### 1.2 LBCL

Anti-CD19 CAR-Ts, axicabtagene ciloleucel (axi-cel) (21), tisa-cel (22), and lisocaptagene maraleucel (liso-cel) (23), showed impressive activity in the third and higher treatment line in a non-comparative clinical trial.

ZUMA−1 is a multicenter, single−arm, registrational phase 1/2 study of axi−cel in patients with refractory LBCL after ≥2 lines of therapy (24). Long−term results from ZUMA−1 demonstrated sustained overall survival (OS), with a median of 25.8 months and a 5−year estimate of 43%. Initial assessments in ZUMA−1 suggested axi−cel may be curative for a subset of patients (25, 26).

In the primary analysis of the pivotal JULIET trial of tisa-cel, the best ORR was 52% and the CR rate was 40% in adult patients with RR LBCL (22). At a median follow-up of 40.3 months, tisa-cel continued to show durable activity (27). The safety profile analysis did not show any secondary tumor, and no deaths were attributed to tisa-cel.

Liso-cel demonstrated significant efficacy with a manageable safety profile as third-line or later treatment in patients with RR LBCL (23). After 2-year follow-up, the ORR was 73% and CR rate was 53% with no new safety signals (28).

Axi-cel and liso-cel showed a significant improvement in PFS and a strong trend in OS in two phase III clinical studies in high-risk RR LBCL compared with salvage therapy (ST) followed by autologous stem cell transplantation (ASCT) (29–32). Kersten et al. reported an updated efficacy and safety results from the primary OS analysis among ZUMA-7 patients aged ≥65 and ≥70 years (33). No new treatment-related deaths occurred. One patient died for an acute myeloid leukemia.

ZUMA-12 is a phase 2, multicenter, single-arm study of axi-cel as part of first-line treatment in patients with high-risk LBCL (34). In the updated analysis with a median follow-up of ≥40 months (35), axi-cel confirmed a high rate of durable responses and no new safety signals. There were 8 deaths due to progressive disease (n=5) and other causes not related to axi-cel (1 COVID-19, 1 esophageal adenocarcinoma, 1 septic shock on Days 350, 535, and 287 post axi-cel infusion, respectively). Two of the 8 deaths (1 progressive disease and 1 esophageal adenocarcinoma) occurred after the primary analysis.

Current guidelines indicate that CAR-T is the standard of care in DLBCL patients with refractory disease, early relapse after first-line chemotherapy, and in third line of treatment (36).

Crombie et. presented the results of patients who were followed in the CIBMTR Cellular Therapy Registry after infusion with commercial liso-cel for the treatment of R/R LBCL (37). Of the 396 patients evaluated, 14 patients developed a second malignancy (5 squamous cell skin malignancy; 3 myelodysplasia; 2 basal cell skin malignancy; 2 a gastrointestinal malignancy; 1 melanoma; 1 myeloproliferative neoplasm, respectively.

In LBCL patients not intended to receive ASCT after failure of first-line therapy outcomes have been poor (38, 39). The phase 2 PILOT study evaluated the efficacy and safety of liso-cel in this setting (40). Sehgal et al. reported the final analysis after 24 months of follow-up or study discontinuation (41). There were no new safety signals. Two patients developed a second primary malignancy (squamous cell carcinoma of skin and malignant external ear neoplasm, n = 1; myelodysplastic syndrome, n = 1).

Ghilardi et al. reported a T cell lymphoma occurring 3 months after CAR-T infusion for non-Hodgkin B cell lymphoma (42). The T cell clone was identified at low levels in the blood before CAR T infusion and in lung cancer. Moreover, the authors evaluated the risk of secondary primary malignancy after commercial CAR-T therapy in 449 patients treated in a cancer center in the United States. Sixty-teen patients (3.6%) had a secondary primary malignancy, and the 5-year incidence was 2.3% for hematological malignancies, and 15.2% for solid tumor. Overall, one case of T cell lymphoma was observed, suggesting a very low risk of this disease after CAR T.

### 1.3 FL

Tisa-cel is approved for adults with R/R FL in the ≥3 rd-line setting (43). The primary analysis of the Phase II ELARA trial reported high response rates and excellent safety profile in extensively pretreated patients with r/r FL (44). Findings from a longer-term update of the ELARA trial continue to demonstrate high response rates and durable remissions with a favorable safety profile (45). No new safety signals were reported. Two patients experienced a secondary malignancy during this longer-term follow-up (squamous cell carcinoma and bladder transitional cell carcinoma). Additionally, 3 new deaths occurred during this updated 2-year follow-up period (progressive disease, n=1; serious adverse events, n=2 (urothelial bladder carcinoma and graft-vs-host disease following allogeneic SCT). None of the malignancies or deaths were considered related to study treatment.

Beyond the potential efficacy of CAR-T in FL, axi-cel results in a high number of durable responses in the third and higher treatment line in a non-comparative clinical trial (46). Axi−cel was tested on the ZUMA−5 multicenter, single−arm, Phase 2 study, in patients with R/R indolent non−Hodgkin lymphoma (NHL), including FL and marginal zone lymphoma (MZL) (47). After a median follow−up of ≥3 years, median PFS was 40.2 months in patients with FL and not reached in those with MZL (25). After ≥4 years median follow−up in ZUMA−5, axi−cel demonstrated continued durable responses and long−term survival in patients with RR iNHL (47). After the 3-year data cutoff date, 1 patient with FL developed a myelodysplastic syndrome, and 1 patient with MZL an acute myeloid leukemia.

### 1.4 MCL

Brexu-cel is an autologous anti-CD19 CAR T-cell therapy approved in adults with R/R MCL following ≥2 prior therapies, including ibrutinib a BTKi (48). After three years in ZUMA-2, brexu-cel demonstrated an ORR of 91%, and a CR rate of 68%. ZUMA-18 is an expanded access study of brexu-cel for the treatment of patients with R/R MCL, including BTKi-naive patients who received ≥ 1 prior therapy following ZUMA-2 enrollment completion. Goy et al. reported the primary analysis of ZUMA-18 and the 4-year follow-up of ZUMA-2 (49). Consistent with ZUMA-2 findings, brexu-cel demonstrated a high level of efficacy, with an ORR of 87%.

### 1.5 MM

Newer treatment options for patients with triple-class RRMM include classes of drugs targeting BCMA, among which are the CAR-T cells idecabtagene vicleucel (ide-cel) and ciltacabtagene autoleucel (cilta-cel) (50).

The first commercially available CAR-T therapy was ide-cel, approved for patients with RR disease after at ‗three lines of therapies, including a proteasome inhibitor, an immunomodulatory agent, and an anti-CD38 antibody (51, 52). In the phase 2 KarMMa trial, at a median follow-up of 24.8 months, patients who received ide-cel showed an ORR of 73% and a CR or better rate of 33% (53). The phase III KarMMa-3 randomized controlled trial results were published comparing ide-cel with SOC (54). No second cancers have been reported.

Cilta-cel was the second CAR-T cell therapy to enter the market for MM and was approved by the FDA and the EMA for the same indications as ide-cel (54–57). At the 28-month median follow-up, a total of 20 secondary tumors had occurred in CARTITUDE-1 and were all considered unrelated to cilta-cel. Six cases of skin cancers were reported, including squamous cell carcinoma (n = 4) and basal cell carcinoma (n = 2). Ten cases of hematologic secondary tumors were reported in ten patients, including low-grade B-cell lymphoma (n = 1), myelodysplastic syndrome (n = 6), and l acute myeloid leukemia (n = 3). One case each of prostate cancer, malignant melanoma, adenocarcinoma, and myxofibrosarcoma also occurred. The accumulation of late-onset malignancies is not unexpected with long-term follow-up of patients with RR MM, many of whom have received subsequent anti-myeloma therapy. Initially referred to as LCAR-B38M, this construct was first studied in humans in a phase I trial (58). Four-year follow-up results were recently published, representing the most extended follow-up to date of any CAR-T therapy for MM. Second primary malignancies occurred in 4 patients [lung cancer (n = 2) and 1 case each of esophageal and cervical cancer].

### 1.6 Genotoxicity and secondary malignancies

The use of retroviral and lentiviral vectors for lymphocyte transduction results in the so-called risk of insertional mutagenesis (59–61).

Although this risk, no genotoxicity of gene transfer into differentiated cells, including T cells, has been reported, and. no vectors-related transformational events in more than 500 cumulative follow-up years of patients treated with engineered T cells have been observed (62). Pre-existing mutations in patients receiving CAR-T cell therapies can result in secondary malignancies in some cases (63). A recently published study showed a clonal hematopoiesis before treatment in 86% of patients receiving CAR-T cells, among those with prolonged cytopenias (64).

The risk of second malignancies in the context of CAR-T therapies needs to be evaluated against other available therapeutic approaches (65). The development of secondary malignancies is demonstrated after radiotherapy and/or chemotherapy treatment. The genotoxicity of these treatments is a well-known long-term side that results in a predisposition to the development of neoplasms (66).

Increased risk of second cancer after stem cell transplantation (SCT) ranges from 8 to 28% (67). Incidence correlates with the type of therapy given before and after SCT, the age of the patient, and the type of transplantation. Most of these secondary malignancies are myeloid-derived.

## 2 Discussion

CAR-Ts have revolutionized the treatment of some hematologic cancers. The development of a second cancer was considered a significant potential risk at the time of approval and is included in the risk management plan. Close monitoring was provided, with a requirement to conduct long-term follow-up studies on safety and efficacy and to submit safety update reports. The reported incidence of second cancers is negligible given the global number of people worldwide who have received CAR-T therapy. It should not be forgotten that the alternative to CAR-Ts is chemotherapy combined with cytotoxic agents with non-negligible secondary malignancy rates (16, 68–72). The true nature and frequency of secondary malignancies are yet to be confirmed. Important patient characteristics such as age, prior immune status, whether the CAR gene is present in the tumor, the landscape of genetic mutations carried by patients in these cases, and other clinical features such as the time from CAR T infusion to the development of T cell lymphoma, are unknown. Only expanded use in thousands of people can give us an accurate estimate of the risks. However, we must be cautious not to over-emphasize standard practice and not to create alarmism about therapies that save many lives. Indeed, it should be remembered that there have been very few cases of secondary cancers compared with thousands of patients treated and who had no other treatment options. The concern is related to genetically modifying T cells taken from the patient. We use a viral vector that carries a gene inside the T cell to prepare CAR-T cells. The virus integrates into the DNA in only a partially controlled way, so there is a risk that it could damage genes crucial for cell proliferation and differentiation. These genes, when altered, can generate tumor development. This potential risk, known as insertional mutagenesis (73), is therefore linked to the very fact of genetically modifying a cell and has already been observed in other gene therapies, for example, in the treatment of immunodeficiencies (74). The regulator’s website states that the EMA is thoroughly investigating the content of some reports of T-cell neoplasms in patients previously treated with CAR-T cells. The initial approvals of CAR-Ts were subject to the fulfillment of post-marketing requirements, which require observational safety studies to be conducted to calculate the risk of treatment-related adverse events; thus, these are safety-focused trials to be conducted on an expanded cohort of people and include an assessment of the risk of secondary malignancies occurring. It is now well known that patients receiving CAR-Ts will need to continue to be monitored within follow-up programs because the occurrence of new, potentially severe adverse events can also be realized over the long term. Nonetheless, it should be noted that correlation with treatment must be proven beyond reasonable doubt, and this is a process that takes time and effort. It must be ascertained that other factors are not contributing and that the disease is not related, instead, to the heavy chemotherapy regimens or frail state of the treated cancer patients. For this reason, extreme caution is needed, both in analyzing reports and communicating their results, to avoid generating unfounded fears to fuel mistrust of therapies now considered lifesaving.

## Author contributions

MMa: Investigation, Supervision, Writing – original draft, Writing – review & editing. GaP: Investigation, Writing – original draft. GiP: Investigation, Writing – original draft. CAl: Investigation, Writing – original draft. BL: Investigation, Methodology, Writing – original draft. MMi: Investigation, Methodology, Writing – original draft. CAr: Investigation, Writing – original draft. MA: Investigation, Writing – original draft. TM: Investigation, Writing – original draft. DL: Supervision, Writing – original draft. VD: Methodology, Supervision, Writing – original draft. MR: Methodology, Supervision, Visualization, Writing – review & editing. FCo: Methodology, Supervision, Visualization, Writing – original draft. FCa: Investigation, Supervision, Writing – original draft. VN: Investigation, Supervision, Writing – review & editing. GF: Methodology, Supervision, Writing – original draft. AI: Supervision, Visualization, Writing – review & editing. MP: Supervision, Validation, Writing – original draft, Writing – review & editing.
